# Digital phenotyping correlates of mobile cognitive measures in schizophrenia: A multisite global mental health feasibility trial

**DOI:** 10.1371/journal.pdig.0000526

**Published:** 2024-06-28

**Authors:** Asher Cohen, Devayani Joshi, Ameya Bondre, Prabhat Kumar Chand, Nirmal Chaturvedi, Soumya Choudhary, Siddharth Dutt, Azaz Khan, Carsten Langholm, Mohit Kumar, Snehil Gupta, Srilakshmi Nagendra, Preethi V. Reddy, Abhijit Rozatkar, Yogendra Sen, Ritu Shrivastava, Rahul Singh, Jagadisha Thirthalli, Deepak Kumar Tugnawat, Anant Bhan, John A. Naslund, Aditya Vaidyam, Vikram Patel, Matcheri Keshavan, Urvakhsh Meherwan Mehta, John Torous

**Affiliations:** 1 Division of Digital Psychiatry, Department of Psychiatry, Beth Israel Deaconess Medical Center, Boston, Massachusetts, United States of America; 2 Sangath Bhopal Hub, India; 3 Department of Psychiatry, National Institute of Mental Health and Neurosciences, Bengaluru, Karnataka, India; 4 Department of Psychiatry, All India Institute of Medical Sciences (AIIMS) Bhopal, Bhopal, India; 5 Department of Global Health and Social Medicine, Harvard Medical School, Boston, Massachusetts, United States of America; 6 National Institute of Advanced Studies, Bangalore, India; Iran University of Medical Sciences, IRAN (ISLAMIC REPUBLIC OF)

## Abstract

Traditional cognitive assessments in schizophrenia are time-consuming and necessitate specialized training, making routine evaluation challenging. To overcome these limitations, this study investigates the feasibility and advantages of utilizing smartphone-based assessments to capture both cognitive functioning and digital phenotyping data and compare these results to gold standard measures. We conducted a secondary analysis of data from 76 individuals with schizophrenia, who were recruited across three sites (one in Boston, two in India) was conducted. The open-source mindLAMP smartphone app captured digital phenotyping data and Trails A/B assessments of attention / memory for up to 12 months. The smartphone-cognitive tasks exhibited potential for normal distribution and these scores showed small but significant correlations with the results from the Brief Assessment of Cognition in Schizophrenia, especially the digital span and symbol coding tasks (r2 = 0.21). A small but significant correlation (r2 = 0.29) between smartphone-derived cognitive scores and health-related behaviors such as sleep duration patterns was observed. Smartphone-based cognitive assessments show promise as cross-cultural tools that can capture relevant data on momentary states among individuals with schizophrenia. Cognitive results related to sleep suggest functional applications to digital phenotyping data, and the potential of this multimodal data approach in research.

## Author summary

While cognition is a well-known driver of functional disability in illnesses like schizophrenia, assessing cognition in routine clinical care is challenging because current assessments require expert training and can require at least half an hour to complete. Likewise, factors that we know impact cognition like sleep are also challenging to assess and so rarely integrated or discussed in care settings. In this pilot study, we explored how smartphones may help overcome both these challenges by enabling personalized and real-time data on cognition and sleep. This study recruited 76 people with schizophrenia at sites in the United States and India and asked them to use the open-source mindLAMP app to facilitate data capture of cognition through completing app-based exercises and sleep by allowing the collection of phone-based sensors (accelerometer and screen-state). Results showed that this smartphone data collection was feasible across these global study sites and that there was a correlation between sleep and cognition, suggesting potential for using this method to help guide better understanding and care in schizophrenia.

## Introduction

Cognitive impairment in schizophrenia is associated with greater functional impairment than other aspects of the illness (i.e., hallucinations) [1]. Yet assessing cognition remains infeasible in the course of routine care in most settings as gold standard assessments can take up to 90 minutes to complete and require specialized training [2]. This means that cognitive testing is often expensive and inaccessible to people who need it the most. The need for new approaches for assessing cognition has spurred interest in digital approaches and computerized assessments. Yet, less research has focused on the subset of computerized assessments delivered on smartphones despite smartphones being more accessible than computers to patients with schizophrenia[3,4], prevalent in global mental health settings, and able to capture parallel functional data with no additional effort or cost.

The potential of remote cognitive assessment in schizophrenia is increasingly discussed and well outlined in a recent scoping review [5]. This review highlighted that smartphone cognitive assessments are a recognized category of remote cognitive assessments and highlighted feasibility of data collection from early pilot studies [5]. Yet, this review also noted that a primary barrier to advancing research in this area is the lack of standardized scoring for smartphone-based cognitive assessments, which impedes replication and interpretation of results. Thus, in this paper, we focus on normative metrics, real-world validity, and the global health potential of smartphone-based cognitive assessments in schizophrenia.

While the traditional goal of any cognitive task is to isolate and measure a specific cognitive process, the goal with smartphone-based assessments can also be expanded. Like traditional assessments, smartphone-based assessments also seek high discriminatory power to measure variability in patients’ performance. However, the assessment of validity in the context of smartphone-based assessments extends beyond correlation to traditional tests with potential additional value in measuring relevant environmental, behavioral, and psychological factors gathered simultaneously from the smartphone. For example, it is possible to obtain an attention/memory score from a smartphone assessment that may be comparable to a paper/pencil test, but the smartphone also has access to data on that patient’s recent sleep patterns, home time, and mood symptoms, which may add important context towards interpreting the results. A 2024 review of smartphone-based cognitive assessments in schizophrenia highlighted the feasibility of this approach across numerous cognitive domains, noting that the integration of smartphone sensor data may provide important environmental, social, and behavioral context to help interpret results [5].

However, this flexibility and potential of smartphone assessments also creates challenges around reliability. As reliability is a measure that reflects interactions between the task, participants, and the context in which they complete the task, it assumes patients are assessed in a similar state and context. Given that smartphone-based assessments are assessing participants longitudinally via their smartphones across their daily lives, they will be in different mental and cognitive states as well as different settings in the place of contexts (i.e., home, work, school, noisy environments, etc.).

The challenges of smartphone-based assessments can, however, be reframed as unique advantages, considering that validity beyond direct correlations to traditional cognitive assessments allows better application of the multidimensional (i.e., smartphones can also capture measures of sleep, steps, mood) and longitudinal (i.e., days, or even years) data that is impossible to measure with classical methods. Often referred to as digital phenotyping, the use of smartphone sensors to passively capture clinically relevant metrics of behaviors, environments, and social interactions adds a rich new dimension of data relevant to furthering our understanding of cognition. With this richer data, new digital smartphone assessments may serve as novel metrics in new multimodal biomarkers of risk, diagnosis, prognosis, or treatment response. A second reframing of reliability is to consider results beyond traditional between-person metrics and now as within-person and personalized metrics of change. This approach allows clinicians to use results to inform clinical decision-making and offer patients personalized interventions.

To better explore this potential, in this paper, we extend prior research on attention/memory smartphone assessments for schizophrenia with a new focus on context derived from smartphone sensor data. A 2022 review of remote assessments of cognition in schizophrenia noted that the Jewel Trails Making Task on mindLAMP showed evidence of being valid and reliable [6]. Yet this review also identified the chief weakness of the remote cognition space, i.e., the need for normative data and scoring metrics to enable validation and replication. Furthermore, there is a need to support the replication of these findings across different contexts and cultures, representing the unique potential of digital health tools like smartphone apps, yet one that is seldom explored.

There have been several impressive smartphone-based studies exploring cognition, but the focus has largely been on finding correlations between individual smartphone assessments and their gold-standard counterparts. For example, prior studies have reported high correlations between smartphone-based metrics related to emotion recognition [7], the Stroop and letter-verbal fluency test [8], Hopkins Verbal Learning Test [9], facial recognition affect [10], and Trails A/B test [11–13]. Building off these impressive results, we now explore the role of smartphone-based assessments of cognition beyond feasibility and across diverse settings in the United States and India. We hypothesized that the app-based Trails A/B test would most highly correlate with the clinically administered Digital Symbol Test among assessments in the Brief Assessment of Cognition in Schizophrenia given both assess aspects of motor speed, attention, and visuo-perceptual function. Extending prior research on these tests, which has found modest outcomes with functional outcomes [14–15], we hypothesized that patterns of sleep duration as captured with digital phenotyping would strongly correlate with app-based Trails A/B scores. This is because attention and working memory in schizophrenia, both domains assessed by the Trails A/B, are likely directly impacted by poor sleep [16].

Thus the primary two goals of this paper are identifying potential normative metrics of smartphone-based cognitive assessments and exploring the association between smartphone-derived measures of sleep cognitive assessments. Given this study was conducted across three unique sites across the United States and India, assessing the feasibility of cross-cultural use of smartphone-based cognitive assessments in schizophrenia is the third goal.

## Methods

### Study overview

Smartphone-based cognitive assessments were an exploratory aspect of the Smartphone Health Assessment for Relapse Prevention (SHARP) study. The study protocol and detailed participant descriptions are already published [17–19]. The research was conducted across three distinct sites: Boston, MA, USA; Bhopal, India; and Bengaluru, India. The study comprised two participant groups– 76 individuals diagnosed with schizophrenia and in treatment and 56 healthy controls with recruitment methods and demographics of included participants already published [18] These participants were actively engaged for a duration of up to 12 months, during which they underwent a combination of monthly in-person or telehealth assessments and utilized the mindLAMP app to capture digital phenotyping at all times and cognitive data up to twice a week. Only data from those participants with schizophrenia were used for this particular analysis.

### Participant characteristics

Participants aged 18 and older with a diagnosis of schizophrenia, within 5 years before September 2021, were selected based on established DSM diagnostic criteria and assessments. The only exclusion criteria were patient or clinician concern about partaking in the study. Detailed participant demographics, including age, sex, education, ethnicity, and race are presented below, see Table 1, and available in previous literature [20].

**Table 1:** Participant Demographics from the SHARP Study only include those participants with a schizophrenia diagnosis.

		Number of Participants
Race	White/Caucasian	16
	Black/African-American	5
	Multiracial or other	4
	Asian	50
	Missing	1
Ethnicity	Hispanic or Latino	5
	Not Hispanic or Latino	70
	MIssing	1
Education	Eighth grade or less	3
	Some High School	5
	High School Graduate/GED	12
	Some College	31
	4-year College Graduate or Higher	24
	Missing	1
Sex	Male	41
	Female	33
	Other	2

### Institutional review board approval

Ethical clearance was obtained from the Institutional Review Boards (IRBs) of each site: Beth Israel Deaconess Medical Center, Sangath IRB, All India Institute of Medical Sciences Bhopal Institutional Human Ethics Committee (IHEC), and the National Institute for Mental Health and Neurosciences IHEC. Written informed consent was obtained from all participants. Given the nature of data collected in the study including geolocation, participants were guaranteed that their data would not be shared outside of the study team.

### Assessments and data collection

In-person (or telehealth) assessments included the administration of well-established measurement tools at monthly intervals: the Brief Assessment for Cognition in Schizophrenia (BACS) for cognitive evaluation20, the Positive and Negative Symptoms Scale (PANSS) to gauge psychosis severity [21], the Pittsburgh Sleep Quality Index (PSQI) to quantify sleep patterns [22], and other metrics not related to this paper. The BACS [23] and PANSS [24,25] which are the focus of this study have been used before and validated in Indian populations. The BACS offers a composite score that is based on an evaluation of working memory, verbal memory, fluency, attention, processing speed, and executive function. The PANSS also offers a composite score that is based on an evaluation of positive, negative, and general symptoms.

While these assessments were collected monthly, as outlined below, the smartphone assessments were offered more frequently between these monthly study visits.

### mindLAMP data collection

mindLAMP is an open-source software platform that enables personal smartphones (Apple and Android) to serve as digital data collection devices capturing real-time data on behaviors like sleep, step count, and screen time. At the time of this study, mindLAMP was the only platform available to capture both digital phenotyping and cognitive assessment data. mindLAMP collects survey and sensor data customized by type and frequency for each unique research protocol or clinical case [17]. It allows the sharing of this data back with the user and can be configured to offer on-demand or triggered psychoeducation or therapeutic interventions. Across a series of focus groups with patients, family members, and clinicians, the mindLAMP app was culturally adapted for use in India 26. While the data collection (sensors and translated surveys) remained the same across all versions of the app used in this study, the language, images, and psychoeducation content were all adapted [26]. Across all sites, on the smartphone app, participants were asked to complete two short surveys per day, randomly selected from a pool of six surveys regarding mood, anxiety, medication adherence, sleep, sociability, and psychosis. Twice a week, participants were also offered optional smartphone-based cognitive assessments, as outlined below. The smartphone app also collected passive sensor data at all times. This was used to derive metrics related to time spent at home per day, mobility entropy (a measure of the routine of the timing of locations visited compared to prior days) per day, screen duration per day, and steps per day. Screen state data and accelerometer data were both used to derive the duration of phone inactivity per day, which was used to approximate sleep duration.

### Smartphone-based cognitive assessments

**Fig 1:**
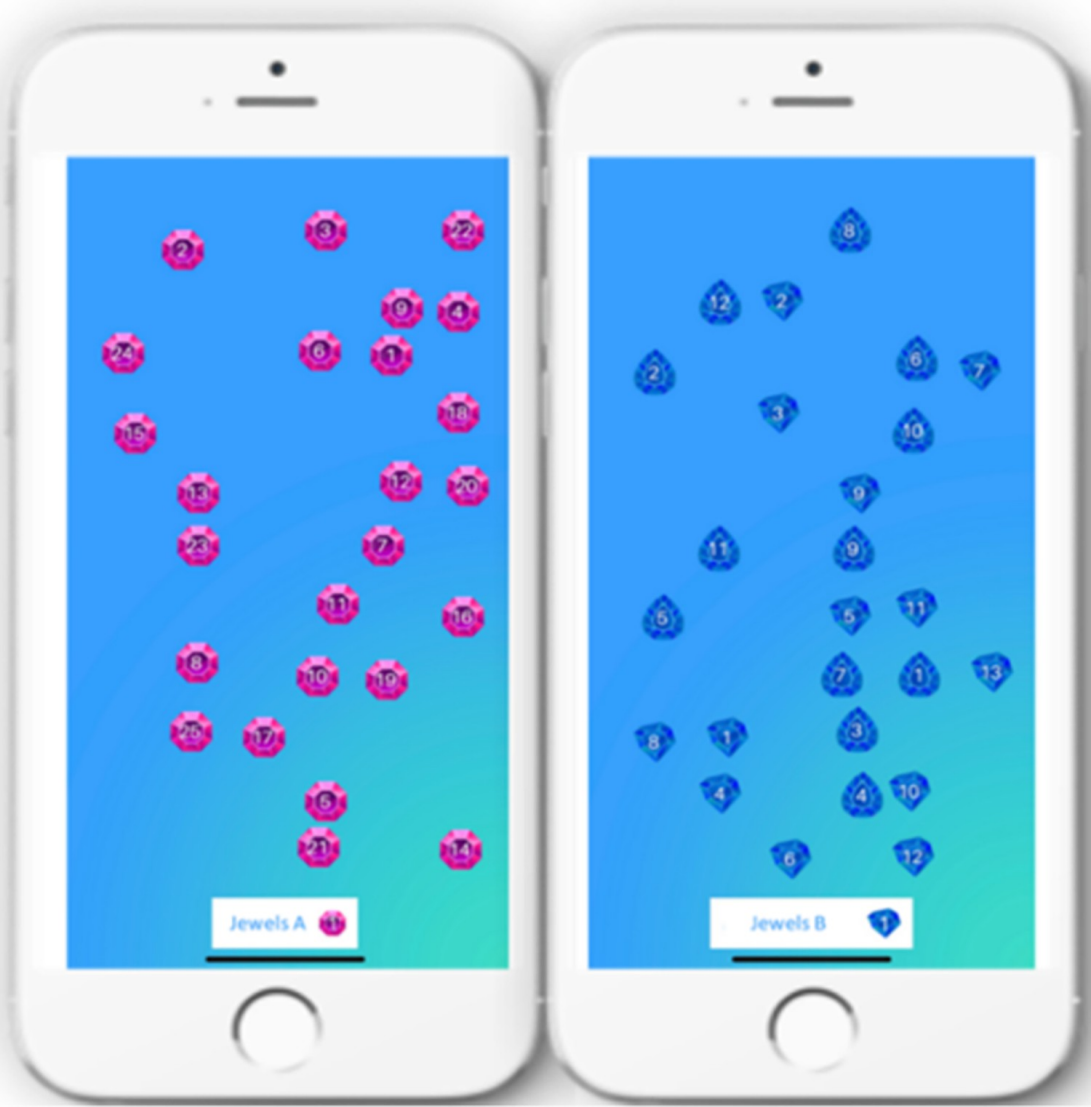
Screenshots of the Jewels A (left) and Jewels B (right) assessments on the mindLAMP smartphone app.

Participants were prompted to complete smartphone-based cognitive assessments, which included modified Trails A (called Jewels A) and Trails B (called Jewels B) tasks up to twice a week. Trails A and B are well-established general cognitive functioning tests that assess a participant’s working memory, visual processing, visuospatial skills, selective and divided attention, processing speed, and psychomotor coordination. There was no compensation tied to engagement. As reported in past papers [13,14], these assessments offered on the smartphone are modified in the following manner: 1) a new assessment is randomly generated with each use, 2) patients only need to tap the target instead of drawing a line to it, 3) the order of the targets may involve crossing a line (if one was drawn). Different shapes present alternative targets in the Trails B version of the task (see Fig 1 below). Validation of these smartphone-based versions against gold standard assessments has been conducted in prior papers for the Trails A (r  =  0.57) and Trails B (r  =  0.58) assessments [12]. Participants were shown how to use these app-based cognitive tests, on their own smartphone, during study onboarding.

### Statistical analysis

Given the emphasis of the current analysis on understanding the cognitive tests, and that participants were asked to complete these assessments as quickly as possible but with the least number of errors, we opted to score these with rate-corrected scores [27], which best reflect speed and accuracy. This was calculated by dividing the number of correct Jewels picked by time taken. This helped account for individuals who exited the game prematurely. Higher scores meant better performance, reflecting quick yet accurate task completion. Code for analyzing mindLAMP data is freely accessible at: https://docs.lamp.digital/data_science/cortex/what_is_cortex/

First, we estimated participant engagement in these activities by calculating our active data quality, i.e. the number of surveys received divided by the number of surveys we expected to receive, had all participants filled out all requested surveys. Second, the distribution of the rate-corrected scores was then tested for normality using D’Agostino and Pearson’s omnibus test for normality, and a quantile-quantile plot was generated to provide a visual representation of the normality of the data [28,29]. Third, to examine the linear correlation between the mean Jewels A/B scores and the mean subscores of the BACS cognitive assessments for each participant, regression plots were constructed along with coefficients of determination (R2) and 95% confidence intervals. Fourth and finally, the correlation between phone inactivity per week (utilized as an (over)estimate of sleep duration) and Jewels A/B scores from the same week was measured.

## Results

**Fig 2:**
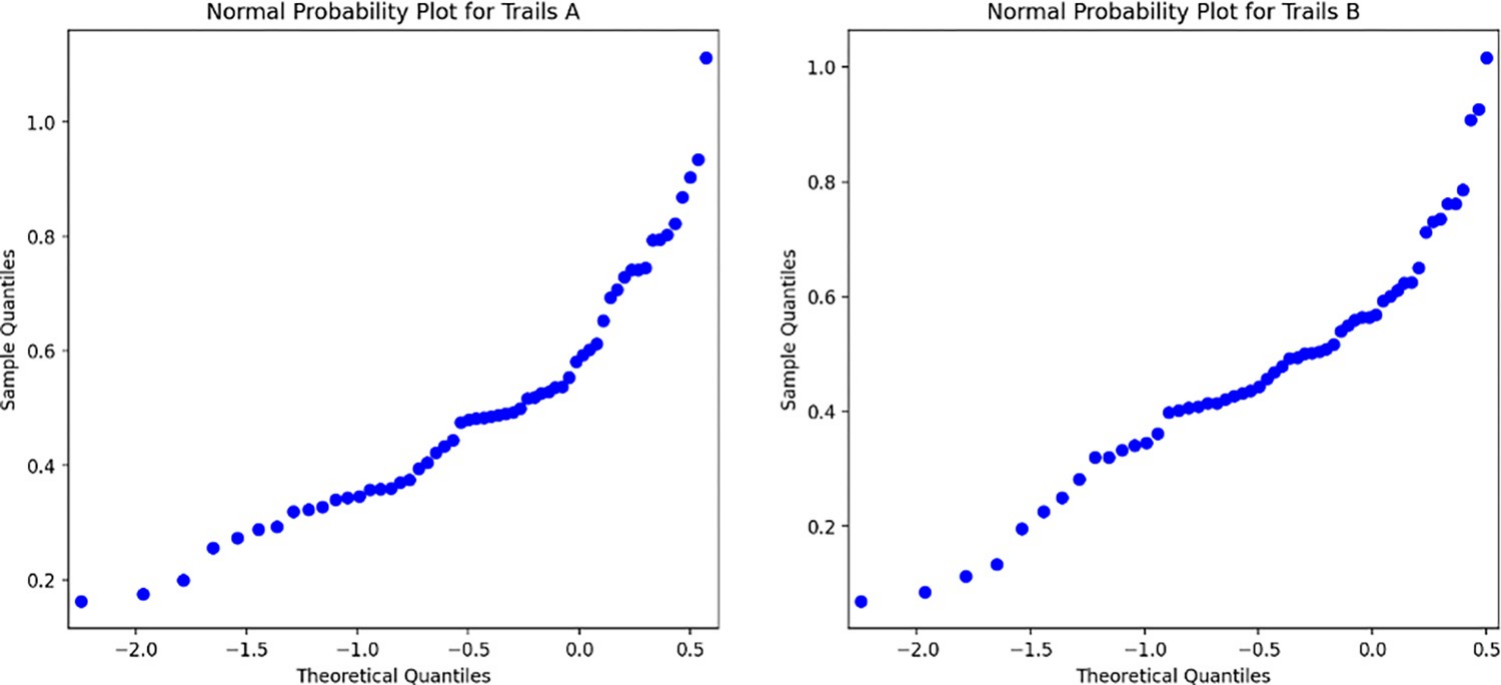
Quantile-quantile plot for Jewels A (left) and Jewels B (right).

Engagement for these voluntary cognitive assessments over 12 months for the Jewels A task was 31.2% and for the Jewels B task was 29.5%. The null hypothesis that the distribution of Jewels A and Jewels B participant scores came from a normally distributed parent population failed to be rejected for both Jewels A (p = 0.14) and Jewels B (p = 0.53) (Fig 2).

**Fig 3:**
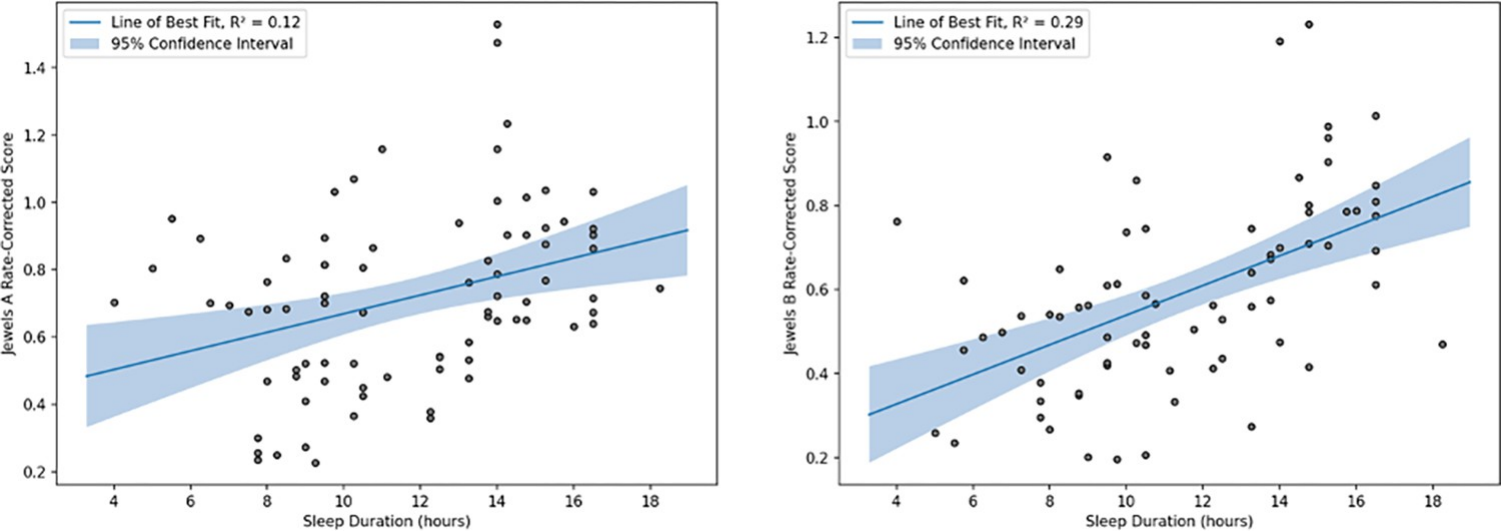
Correlation between smartphone sensor estimated sleep duration and Jewels A(left) and Jewels B(right). Results show a positive correlation between more sleep and higher scores on both smartphone assessments, reflecting better performance.

There was a modest positive association between Jewels A/B scores and the subscores of the BACS cognitive assessments with R2 values ranging from 0.00 and +0.24 (Fig 3).

**Fig 4:**
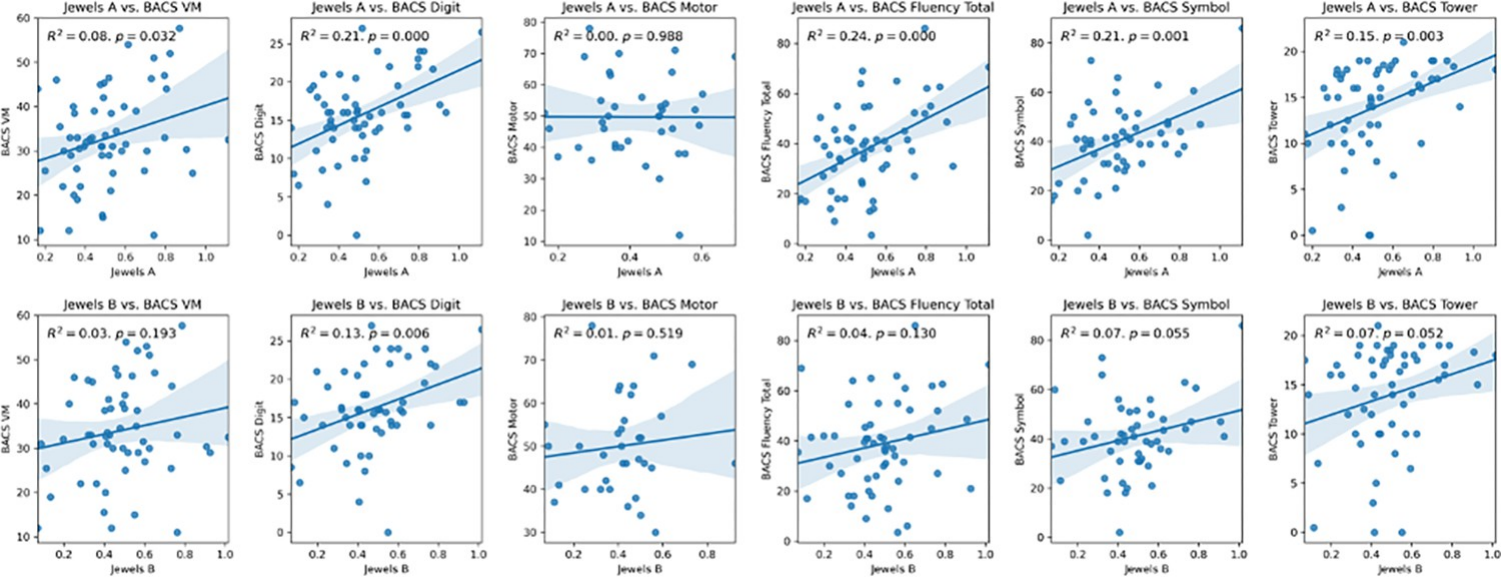
Correlation between the Jewels A/B scores and BACS cognitive assessments subscores. Note: Token motor task was not administered at the Boston site because of complete remote administration. Abbreviations: VM- Verbal memory, Digit- Digit sequencing, Motor- Token Motor, Symbol- Symbol coding, Tower- Tower of London. High levels of variation are notable in the distribution of scores on both the BACS scores and Jewels A/B smartphone scores.

Finally, there was a statistically significant positive linear correlation between sleep duration, inferred from phone activity patterns, and higher Jewels A/B scores (Fig 4).

## Discussion

Our results demonstrate a feasible methodology for scoring smartphone assessments related to attention/memory and demonstrate that the scores themselves may be normally distributed, with correlations to functional outcomes related to sleep duration. While prior studies have focused on correlations to gold standard metrics, results here suggest that the smartphone version of the Trails A/B tasks may fit a normal distribution. The positive correlations between the smartphone scores of Jewels B task and the related BACS scores among participants with schizophrenia, strongest for tests of attention (digital symptom and digit sequencing) than motor function is expected. The higher correlations for Jewels B task, as compared to the simpler Jewels A task, to sleep duration patterns suggest an association between cognition and sleep.

While our results can only be understood in the context of pilot results, the correlations between app-based cognitive scores and health outcomes related to sleep suggest the potential for functional correlates. These results make sense as the relationship between sleep and cognition is well established, but being able to assess both simultaneously from a patient’s own smartphone could enable more personalized care based on lifestyle factors. There are also implications for formal regulatory approval, digital health tools often need to show not only score-driven outcomes but also functional-driven outcomes as well. This is in line with the US FDA’s Framework for the Use of Digital Health Technologies in Drug and Biological Product Development that notes the agency seeks both verification and validation [30]. Smartphone-based tools that can capture new data like cognitive assessments, but also digital phenotyping outcomes related to functioning may thus offer a productive platform for ongoing research.

Our results highlight the potential of smartphone-based cognitive assessments in a global mental health context. The use and completion of the tasks were feasible at sites in India and the United States. The cultural adaptation of the app [26] was minimal for these cognitive assessments given their universal nature and lack of reliance on language, metaphors, or imagery. We were not able to statistically compare the results between the three sites given the small sample sizes at the individual sites. While a direct comparison is not possible, engagement with mental health apps over weeks is often far lower (around 10%) [31] suggesting the gamified nature of these tasks may encourage relatively high rates of use. This higher engagement is beneficial as it allows for ongoing monitoring of impairment and changes in function over time.

The potential of digital measures of cognition extends beyond schizophrenia. A 2023 review of repetition in DSM-5 constructs found that difficulty with concentration is the second most repeated symptom and featured in 17 unique diagnoses [32]. Using cross-sectional measures of working memory accuracy and global memory quotient in patients with bipolar disorder and schizophrenia, another recent paper suggested metrics of cognitive impairment may serve as intermediate phenotypes [33] The use of novel cognitive measures to enhance the stratification of mental health disorders thus offers a potential application of this work. But before these cognitive tasks can be used for monitoring disease progression or as diagnostic tools, more work is needed to validate their use against both gold standards and real-world functional outcomes.

While there have been overall fewer studies assessing digital cognitive biomarkers in schizophrenia, in the related field of cognitive biomarkers for mild cognitive impairment and dementias, a 2022 review noted that most digital assessments showed comparable or even better diagnostic performance than traditional paper-and-pencil tests [34]. Our results here in schizophrenia suggest a similar trend. Yet, there are still no smartphone tests, in neurology or psychiatry, with high discriminatory power, validity, and reliability [35]–which are necessary to establish a bar for the state of this research. Our results suggest that potential normative scoring offers progress in this direction as it at least means that study results can be compared to each other and results may reflect meaningful variation. Challenges will include methods able to account for intraindividual fluctuations in self-environment such as noisy spaces and self-reported internal states such as stress can be observed in momentary cognition data [36], which our results show is feasible to capture today with digital phenotyping measures on the environment, [37,38]. Bayesian approach able to use population-level priors and then update those priors with personal information will likely play a critical role in advancing these new digital biomarkers.

### Weakness

One limitation of cognitive assessments is the presence of practice effects. We sought to limit these by designing the smartphone assessments to prevent the formation of task-specific strategies with each task randomly generating new targets and orders upon each new interaction. Prior research using these same app-based cognitive assessments has not noted practice effects [13–15]. A second limitation is that our study had a relatively small sample size, which impacted our ability to examine cognitive differences within each site. Still, the sample is larger than the mean sample size of mild cognitive impairments and dementia focused computerized cognitive assessments [38], suggesting the nascent state of the entire digital assessment space. A third limitation is that these assessments were an optional part of the study meaning that engagement was low. This limitation may mirror real-world engagement patterns with apps and may thus reflect a natural limitation when deploying any digital tools in clinical settings. A fourth limitation is that our study data was collected during the COVID-19 pandemic and the subsequent lockdowns. The sleep data we gathered could potentially reflect patterns influenced by lockdown-related conditions like remote work arrangements, and these patterns may not necessarily extend to situations beyond these limitations. A fifth limitation is that those enrolled in the study possessed high educational achievement which may limit the generalizability to all people living with schizophrenia. A sixth limitation is that we did not record or control for medication status of those enrolled in the study.

## Conclusion

In summary, this study emphasizes smartphone-based cognitive assessments’ potential in establishing normative metrics, demonstrating real-world validity, and their role in global mental health initiatives. The distinct advantages lie in capturing multidimensional, longitudinal data, facilitating novel biomarker development and personalized interventions, with potential for application across diverse settings globally. Ultimately, these findings contribute to understanding smartphone-based cognitive assessments’ utility, paving the way for improved cognitive assessment, intervention, and tailored care in schizophrenia and related contexts.
